# Diurnal variation in corticosterone release among wild tropical forest birds

**DOI:** 10.1186/s12983-016-0151-3

**Published:** 2016-05-04

**Authors:** Philipp Schwabl, Elisa Bonaccorso, Wolfgang Goymann

**Affiliations:** Institute of Biodiversity, Animal Health and Comparative Medicine, University of Glasgow, Graham Kerr Building, Glasgow, G12 8QQ UK; Centro para la Investigación de la Biodiversidad y Cambio Climático, Universidad Tecnológica Indoamérica, Machala y Sabanilla, Cotocollao, Quito, Ecuador; Abteilung für Verhaltensneurobiologie, Max-Planck-Institut für Ornithologie, Eberhard-Gwinner-Str. 6a, D-82319 Seewiesen, Germany

**Keywords:** Corticosterone, Birds, Tropical, Diurnal rhythms, Daily variation, Free-living, Wild, Chocó, Stress

## Abstract

**Background:**

Glucocorticoids are adrenal steroid hormones essential to homeostatic maintenance. Their daily variation at low concentrations regulates physiology and behavior to sustain proper immunological and metabolic function. Glucocorticoids rise well above these baseline levels during stress to elicit emergency-state responses that increase short-term survival. Despite this essence in managing life processes under both regular and adverse conditions, relationships of glucocorticoid release to environmental and intrinsic factors that vary at daily and seasonal scales are rarely studied in the wild.

**Methods:**

This study on 41 passerine species of the Ecuadorian Chocó applied a standardized capture-and-restraint protocol to examine diurnal variation in baseline and stress-related release of corticosterone, the primary avian glucocorticoid. Tests for relationships to relative body mass, hemoglobin concentration, molt status and date complemented this evaluation of the time of day effect on corticosterone secretion in free-living tropical rainforest birds. Analyses were also partitioned by sex as well as performed separately on two common species, the wedge-billed woodcreeper and olive-striped flycatcher.

**Results:**

Interspecific analyses indicated maximum baseline corticosterone levels at the onset of the active phase and reductions thereafter. Stress-related levels did not correspond to time of day but accompanied baseline reductions during molt and elevations in birds sampled later during the September - November study period. Baseline corticosterone related negatively to hemoglobin in the wedge-billed woodcreeper and stress-related levels increased with body mass in the olive-striped flycatcher. There were no substantial sex-related differences.

**Conclusions:**

The results of this study suggest a diurnal rhythmicity in baseline corticosterone release so robust as to emerge in pooled analyses across a highly variable dataset. While this detection in nature is singular, correspondent patterns have been demonstrated outside of the tropics in captive model species. Congruity in daily rhythms and links to physiological and life-history state across disparate taxa and environments may promote the yet unresolved utility of corticosterone release as a global metric for population health. However, certain results of this study also deviate from laboratory and field research at higher latitudes, cautioning generalization. Environmental distinctions such as high productivity and tempered seasonality may precipitate unique life-history strategies and underlying hormonal mechanisms in tropical rainforest birds.

**Electronic supplementary material:**

The online version of this article (doi:10.1186/s12983-016-0151-3) contains supplementary material, which is available to authorized users.

## Background

Glucocorticoids are adrenal steroid hormones integral to physiological and behavioral control in nearly all vertebrate animals. Circulating at low levels in the blood, they serve preeminently as regulators of basic energy balance, directing resource acquisition, deposition and mobilization [1]. Upon perception of a stressor, however, glucocorticoid levels rapidly rise and increase short-term survival probability by eliciting increased cardiovascular tone, hyperglycemia, modified immune activity and enhanced memory consolidation (etc.) while diverting effort from non-essential life processes such as reproduction [2, 3].

Diel rhythms in glucocorticoid activity have been established in mammals and are particularly well characterized in rodent model species [4, 5]. Such rhythms have also been detected early on in birds [6], but consensus here remains rudimentary [7, 8]. Most studies agree in little more than that baseline corticosterone, the primary avian glucocorticoid [9], tends to circulate at maximum levels in early morning hours, often at or just prior to the onset of diurnal activity [6, 10–15]. Even less agreement has taken form on diel rhythms in avian stress-related corticosterone release. Only three avian species have been examined deliberately for diel variation in this trait, with maximum stress-related levels found during the inactive phase in house sparrows [16] and starlings [13]. In contrast, Gambel’s white-crowned sparrows show maximum levels during the active phase [12]. Discord extends also to data on modulation by environmental and intrinsic factors such as photoperiod (e.g. photoperiod-dependent adrenal sensitivity in house sparrows [8] vs. independent responses in starlings [13]) and physiological state (e.g. unexhausted [17] vs. maximally stimulated adrenal capacity to secrete corticosterone in molting house sparrows [8]).

This elementary understanding of rhythmic avian adrenocortical function stems primarily from experimental studies on captive birds [14, 18]. However, it is recurrently emphasized that data from captivity can only partially represent wild avian corticosterone secretion [8, 19, 20], including its diel rhythmicity [12, 19]. Not only are few avian species amenable to laboratory experimentation (with bias towards poultry and other domesticated species [14, 18]), a diminishment of environmental and social cues constrains the range of hormone levels exhibited in captivity [21] and may mask diel change [12].

Field data on avian corticosterone release are particularly lacking for near-equatorial species [22]. Past studies have heavily favored the temperate and arctic zone [23, 24]. In contrast, variation in life-history characters has been mapped much more comprehensively across latitudes and distinctions in several of these traits (e.g. incubation period and clutch size) have been established for the tropics [25]. Given that endocrine mechanisms mediate life-history trade-offs [26], corresponding geographical variation in corticosterone release is likely and merits exploration [22, 27, 28]. For example, as low levels of yolk androgens are coupled to long incubation periods in the tropics [29, 30], low levels of parental baseline corticosterone may relate to small clutches reared here over longer breeding seasons [31, 32]. Implications for conservation biology also promote intensified field research on tropical avian corticosterone release. Adrenocortical performance has become an increasingly popular biomarker of population health [1]. However, widespread dispute over its diagnostic utility and superiority to traditional demographic methods must first be resolved [33–35] with enhanced comprehension of how physiological condition and fitness relate to corticosterone secretion in the wild. These relationships are likely specific and distinct to different environments [33, 36, 37]. It is therefore sensible to prioritize further research on corticosterone release to the most vulnerable and biodiverse regions of the world. The tropical Andes are one such hotspot of conservation priority [38].

In this study we describe diurnal variation in baseline and stress-related corticosterone release in free-living individuals of 41 passerine species of northwest Ecuador. Our results on a diverse array of forest birds of the tropical Andes should forward understanding in a field deficient of data on passerines [16] and from wild birds in general [39], specifically regarding diel patterns of stress-related corticosterone release. We also evaluate the time of day effect on corticosterone variation against the additional predictors relative body mass, hemoglobin concentration, molt status and sampling date in our models. These physiological and seasonal variables represent two prominent correlates of avian corticosterone secretion – body condition [40] and life-history stage [24], the former of which often co-varies with time of day [41]. As such, our study contributes data required to resolve geographic and taxonomic differences in short and long-term avian adrenocortical activity essential to conservation decisions for the Ecuadorian Chocó, one of the most biodiverse regions of the world [42].

## Methods

### Study area

A total of 169 individuals comprising 55 passerine bird species were captured daily between 23 September and 22 November 2014 at Mashpi Rainforest Biodiversity Reserve (0° 9′ N, 78° 51′ W) in the Chocó bioregion of Ecuador near Pacto, Pichincha Province. Rainfall is high throughout the year at this reserve. There are no distinct wet and dry seasons, but interpolated climate records specify April as the rainiest month and place a minimum on July [43]. Sunrise and sunset are medially at 5:56 h and 18:03 h at the site [44]. The sampling period coincided in large part with local breeding activity, as determined morphometrically and based on observations of parental behavior. Although avian breeding phenology remains enigmatic and is unlikely discriminable by region or taxon, corresponding reproductive peaks during the period between September and November have been documented elsewhere in the Ecuadorian Chocó [45–47]. Three of the six sampling sites selected were located at 600 m elevation, separated from one another by ca. 1 km, and three sites were located at 1200 m elevation, also separated by ca. 1 km each. All sites consisted of secondary-growth humid montane forest and were selected based on equable proximity to forest edges, streams and human activity.

### Capture and sampling protocol

Birds were captured predominantly by passive mist-netting (and in two cases using conspecific playback recorded on site). Nets remained under constant surveillance from 6:00 to 16:30 h such that a standardized stress protocol could be applied immediately upon capture. As corticosterone levels may become elevated in the blood within 3 min following a stress stimulus [48], initial blood samples to determine baseline hormone concentrations were collected as quickly as possible via puncture of the brachial vein and capillary withdrawal into heparinized micro-hematocrit tubes. Latency time between a bird’s first contact with the net and withdrawal of ca. 40 μl blood was recorded (median = 2.8 min) and did not exceed 3 min for 93 of 95 samples submitted to laboratory analysis. Birds were then given a numbered color band, weighed to 0.1 g by digital scale (American Weigh Scales, Inc.), measured for tarsus length to 0.1 mm by digital caliper (Mitutoyo America Corporation) and assessed for pre-basic molt in remiges, rectrices and feathers of the head, chest, abdomen and back. Reproductive characters (brood patch and cloacal protuberance) served to assign sex in the absence of plumage dimorphism. Sex was left undetermined for 34 monomorphic individuals. Birds were then placed in an opaque cloth bag until 30 min post-capture, at which time a second, slightly smaller blood sample (ca. 30 μl) was withdrawn to determine acute stress-related (standard bag-restraint) corticosterone release. At this time, a small (ca. 2 μl) aliquot of blood was also assessed for hemoglobin concentration (g/l) with a portable photometric hemoglobinometer (EKF Diagnostic). Plasma for determination of baseline and stress-related corticosterone release was separated from the blood cell fraction immediately after each withdrawal using a battery-powered mini-centrifuge (LW Scientific, Inc.) and then transferred to 250 μl vials. Vials were kept on ice in a portable cooler until the end of each day and then stored frozen at −20 °C.

### Corticosterone analysis

Corticosterone concentrations of 190 plasma samples from 95 individuals comprising 41 species were determined by direct radioimmunoassay (RIA) following [49] and outlined below. As referenced above, this subset was selected based on latency time of the first blood withdrawal (for veracious measurement of baseline corticosterone), with analysis completed in three tiered extraction/assay procedures.

To extract corticosterone, measured plasma volumes (mean = 22 μl) were first vortexed with 10 μl (1500 dpm) labelled corticosterone overnight at 4 °C to allow for equilibration with plasma lipids and binding proteins. Steroid isolation followed by repeated dichloromethane extraction, in which freshly distilled dichloromethane was added to the centrifuged plasma samples, the aqueous phase fixed by freezing in pure ethanol and dry ice and the organic phase decanted and evaporated with nitrogen in a 40 °C water bath. The corticosterone sediment was then left to equilibrate overnight at 4 ° C in 300 μl 0.1 M neutral-pH phosphate-buffered saline (with 1 % gelatin-PBSG). To determine extraction efficiency, an 80 μl aliquot was then drawn from the corticosterone-buffer solution, mixed with 4 ml scintillation fluid (Packard Ultima Gold) and quantified for β-radiation in a Beckman LS 6000 counter alongside total recovery counts prepared prior to extraction.

In proceeding to RIA, each vortexed sample was partitioned into 100 μl duplicates. Positive (25 μl corticosterone standard and 100 μl chicken plasma controls) and negative controls (blanks) were also prepared. The standard curve was generated by serial dilution of 100 μl unlabeled corticosterone in 100 μl PBSG, effectively halving concentrations in each dilution step to generate ten 100 μl standard duplicates ranging from 1000 to 1.95 pg steroid content. Sample duplicates, standard curve duplicates, and controls were then mixed with 100 μl corticosterone antiserum and, after 30 min, treated with 100 μl labelled steroid. In addition to the curve standards, TC (100 μl labelled steroid and 200 μl PBSG), NSB (likewise 100 μl labelled steroid and 200 μl PBSG) and B_0_ (100 μl labelled steroid, PBSG and antiserum) were prepared in 300 μl triplicates to determine total counts, non-specific (background) binding and maximum binding with the antiserum, respectively. All solutions were then left to incubate at 4 °C for 20 h. Finally, bound and free corticosterone was separated at 4 °C via addition of 500 μl dextran-coated charcoal to adsorb all unbound steroid, centrifuged under cooling (4 °C) for 10 min at 4000 rpm, decanted into scintillation vials, mixed with 4 ml scintillation fluid and quantified for β-radiation in the scintillation counter.

Standard curves and endogenous corticosterone concentrations were calculated with Immunofit 3.0 (Beckman Coulter, Inc.). For the three assays, mean (± sd) corticosterone extraction recovery was 88.0 ± 3.0 %, 86.8 ± 3.4 % and 85.0 ± 3.6 %. Lower detection limits were 32.6 pg/ml, 31.2 pg/ml and 30.6 pg/ml, respectively, and all samples were above these limits. The intra-assay coefficients of variation were 5.3 %, 4.4 % and 8.1 %, and the intra-extraction coefficients of variation of a chicken plasma pool were 7.6 %, 9.7 % and 6.8 %, respectively. The inter-assay coefficient of variation among the three assays was 3.5 % and their inter-extraction coefficient of variation was 4.3 %.

### Statistical procedures

Statistical analyses were performed in R version 3.2.3 [50]. To assess diurnal trends in baseline and stress-related corticosterone release, means from birds captured during 6:09 h–8:50 h, 8:51 h–11:06 h, 11:07 h–12:49 h and 12:50 h–16:23 h sampling time periods were first compared by one-way analyses of variance (ANOVA). These 4 categories were chosen to partition the daily capture period into sequential time intervals comprising equal sample sizes (*n* = 23, 23, 23 and 24, respectively) of sufficient statistical power. Each of these intervals incidentally holds one of four bouts of avian activity observed to correspond with daily weather trends at the study site. Birds generally arrived at capture sites discontinuously as members of foraging flocks. These mixed groups often peaked in activity near 8:00 h as rains subsided and again around 12:00 h before winds and temperatures rose into afternoon. A third peak recurred in the hour before dusk. 9:00 h–11:00 h also often coincided with elevated activity under overcast, temperate conditions. Results from the categorization scheme outlined above were then validated by sorting data into four sequential intervals of equal length in time (158 min each) and reiterating ANOVA for differences in mean baseline and stress-related corticosterone release. For inferences from the models we obtained Bayesian posterior parameter estimates and their 95 % credible intervals, employing the *sim* function in the *arm* package [51] over 10,000 simulations with an uninformed prior distribution. Unlike null-hypothesis testing, Bayesian statistics do not provide *p*-values. Instead, meaningful differences between groups are assessed by comparing ranges of 95 % credible intervals. These intervals provide an estimate of 0.95 probability for the group means. If the credible interval of one group does not overlap with the mean estimate of another group, the groups are assumed to differ from each other.

To further evaluate baseline and acute stress-related corticosterone dependence, separate linear regression models were fitted to the corticosterone response variables to test the categorical predictor molt status and the covariates sampling time of day, sampling date, sampling latency, hemoglobin concentration and body mass : tarsus ratio. The latter ratio was replaced by a scaled mass index [52] in intraspecific analyses for which additional mass and tarsus measurements were available from prior studies at the site (unpublished data from C. Morochz). This index may more reliably indicate size of energy reserves and other components of body condition in birds. Values for all covariates were standardized by centering (subtraction of the sample mean) and scaling (division by the sample standard deviation) to z-scores. Regression models were selected by ranking candidate models incorporating one to all combinations of the independent variables by Akaike’s information criterion modified for small sample size (AIC_c_ [53]). As this criterion includes a penalty for each incorporated parameter, encounters with chance correlations by multiple testing were restrained by the method’s direction to balance model fit and size. Substantial support was inferred only for models within 2 AIC_c_ of the first-ranked model (i.e. ∆AIC_c_ ≤ 2) and for which credible intervals of all predictor coefficients did not overlap zero. Retained models were then checked for normality of the error distribution, homoscedasticity of the errors, leverage and linearity in the relationship between predictor and response variables. In consequence, ANOVA and model selection programs were reiterated applying log-transformed response variables.

The AIC_c_ model selection procedures outlined above were first performed across all species to investigate general patterns. Within this interspecific tier of analysis, separate models for males and females were also established. Intraspecific analyses followed for the dataset’s best sampled species, the wedge-billed woodcreeper (*Glyphorynchus spirurus*; *n* = 17) and the olive-striped flycatcher (*Mionectes olivaceous*; *n* = 10). Sex was not partitioned in these intraspecific analyses due to limited sample sizes. In all analyses, baseline corticosterone concentrations were applied directly while acute stress-related changes in corticosterone were applied as relative increases from the baseline concentration: (30 min post-capture corticosterone concentration) – (baseline corticosterone concentration). This latter variable was also applied directly as absolute (gross, independent of baseline) concentrations measured 30 min post-capture, because baseline and stress-related concentrations of corticosterone may be independent and regulate different traits [3, 17]. This measure may additionally serve to rate maximum stress-related corticosterone release capacity and is hereafter offset with quotation marks and termed “stress-induced corticosterone” (in distinction to the relative measure “increase in corticosterone”, also specified in quotes hereafter). Given its independence to baseline concentrations, modeling of “stress-induced corticosterone” disregarded sampling latency as a candidate variable. Accordingly, omission of data from two individuals (one orange-bellied euphonia, *Euphonia xanthogaster*, and one slaty-capped flycatcher, *Leptopogon superciliaris*) sampled for baseline corticosterone later than 3 min post-capture (3.5 min and 4 min latency, respectively) was necessary only in analyses of baseline and “increase in corticosterone”, not in analysis of “stress-induced corticosterone”. Lastly, the *ape* package [54] in R was employed to establish a strict consensus phylogenetic tree from recent avian molecular phylogenies [55] for the species of this study.

## Results

**Fig. 1:**
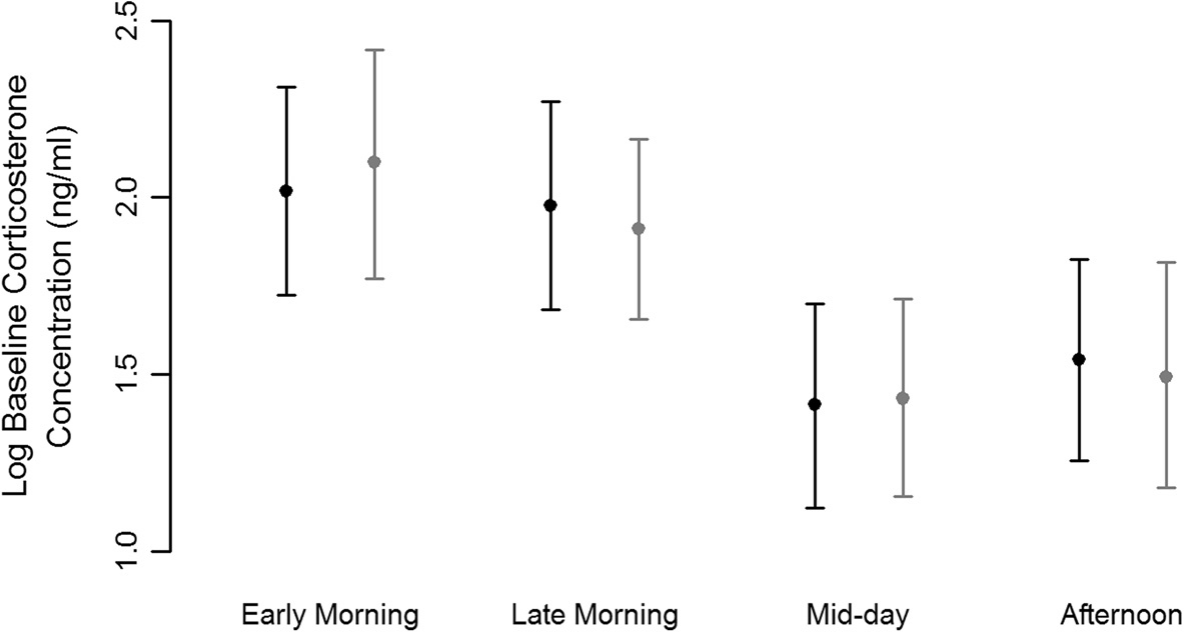
Posterior means of baseline corticosterone measured in blood samples from birds captured at different times of day. Black points denote time intervals selected to partition data into sets of equal sample size (*n* = 23, 23, 23 and 24, respectively). Grey points sort data into sets of equal length in time (i.e. 4 sequential intervals of 158 min each; *n* = 19, 30, 25 and 19). Dispersion bars indicate 95 % credible intervals and suggest corticosterone levels obtained from the first sampling interval (2.018 ng/ml and 2.099 ng/ml) to be higher than those from the third interval (1.417 ng/ml and 1.434 ng/ml). Dispersion of posterior means from the second (1.977 ng/ml and 1.911 ng/ml) and fourth intervals (1.541 ng/ml and 1.494 ng/ml) overlap slightly with others. Log denotes the natural logarithm (to base *e*)

Baseline corticosterone concentrations differed among sampling intervals (ANOVA, F_3,89_ = 4.427), with levels obtained from the first (early morning) and second (late morning) sampling intervals higher than those from the third (mid-day) and fourth (afternoon) intervals. This change in baseline corticosterone over time intervals chosen to aggregate data by equal sample size remained when arranging data for equal length in time (ANOVA, F_3,89_ = 4.611). Again, baseline concentrations from the first and second of these equal-length (158 min) intervals were higher than those from the third and fourth such intervals (see Fig. 1 for Bayesian posterior mean estimates and credible intervals). Sampling time of day also correlated with baseline corticosterone levels in subsequent linear regression analysis (see below). “Increase in corticosterone” and “stress-induced corticosterone” did not vary over the day’s sampling intervals.

AIC_c_ model comparison (Table 1 and Additional file 1: Table S1) identified molt status, sampling time of day and sampling latency as the best predictors of interspecific variation in baseline corticosterone (*r*^2^ = 0.29, F_3,89_ = 12.010). Molting individuals and those sampled later in the day yielded lower baseline levels than non-molting individuals and those sampled earlier in the day. Baseline levels increased with sampling latency (even within the 3 min latency cut-off applied to select samples). In interspecific analysis partitioned by sex, the model selected for males retained the effect of these three predictors yet additionally identified sampling date as a fourth explanatory variable (*r*^2^ = 0.49, F_4,25_ = 6.044), with individuals sampled later in the September - November study period yielding higher levels of baseline corticosterone. Baseline levels in females were also best explained by a negative relationship to the predictors molt status and sampling time of day (*r*^2^ = 0.29, F_2,26_ = 5.234), yet without correlation to sampling latency or sampling date. No model for sex-independent interspecific variation in “increase in corticosterone” met selection criteria. In sex-dependent analyses, however, molt status and sampling date best predicted male “increase in corticosterone” (*r*^2^ = 0.27, F_2,27_ = 9.949), with molting individuals and those sampled at earlier dates showing lower increases than non-molting individuals and those sampled at later dates. Within females, substantial support was not found for any model of “increase in corticosterone”, although the top-ranked model figured a positive trend to sampling date (*r*^2^ = 0.14, F_1,26_ = 4.058). Sex-independent interspecific variation in “stress-induced corticosterone” was best albeit insubstantially explained by molt status (*r*^2^ = 0.06, F_1,93_ = 5.526), with molting individuals yielding lower levels 30 min post-capture than non-molting individuals. In male-specific analysis, sampling date complemented the negative relationship to molt to explain interspecific “stress-induced corticosterone” more substantially (*r*^2^ = 0.26, F_2,28_ = 4.971), with individuals sampled at later dates showing higher levels after 30 min than those sampled at earlier dates. Within females, substantial support was not found for any model of “stress-induced corticosterone”.

**Table 1:** Baseline corticosterone (y_1_), “increase in corticosterone” (y_2_) and “stress-induced corticosterone” (y_3_) models selected from AIC_c_ analyses

	Response Variable	Linear Formula (Intercept and Variable Coefficients)							ω_i_
		Int.	Time	Date	Molt	Hemo.	R. Mass	Lat.	
Total	y_1_	+1.871 [+1.697, +2.046]	−0.236 [−0.388, −0.086]	---	−0.325 [−0.631, −0.016]	---		+0.296 [+0.150, +0.444]	0.29
	y_3_	+4.130 [+3.976, +4.283]	---	---	−0.310 [−0.575, −0.048]	---	---	n/a	0.19
Males	y_1_	+2.179 [+1.846, +2.514]	−0.312 [−0.549, −0.075]	+0.377 [+0.095, +0.646]	−0.714 [−1.324, −0.104]	---	---	+0.278 [+0.026, +0.525]	0.26
	y_2_	+4.303 [+3.963, +4.640]	---	+0.376 [+0.076, +0.676]	−0.838 [−1.436, −0.259]	---	---	---	0.24
	y_3_	+4.396 [+4.087, +4.716]	---	+0.378 [+0.094, +0.665]	−0.757 [−1.319, −0.208]	---	---	n/a	0.36
Females	y_1_^a^	+1.838 [+1.491, +2.198]	−0.356 [−0.641, −0.064]	---	−0.661 [−1.204, −0.108]	---	---	---	0.09
*G. spirurus*	y_1_	+1.952 [+1.591, +2.312]	−0.667 [−1.110, −0.219]	---	---	−0.713 [−1.187, −0.235]	---	---	0.36
	y_3_	+3.525 [+3.171, +3.863]	---	−0.439 [−0.842, −0.042]	---	---	---	n/a	0.21
*M. olivaceus*	y_2_	+4.510 [+4.218, +4.805]	---	---	---	---	+0.366 [+0.057, +0.682]	---	0.51
	y_3_	+4.601 [+4.318, +4.885]	---	---	---	---	+0.346 [+0.048, +0.639]	n/a	0.54

Models are listed with formula and Akaike weight (ω_i_). Coefficient of determination, degrees of freedom and F-ratios are given in the main text. Credible intervals (in square brackets) of all predictor coefficients in these selected models do not overlap zero. This selection criterion was not met by other models within 2 AIC_c_ of top-ranked models (see Additional file 1: Table S1). Corticosterone response variables (ng/ml) are log-transformed to base *e*. Models are given for total and sex-partitioned interspecific corticosterone variation as well as for intraspecific analyses of the wedge-billed woodcreeper (*G. spirurus*) and olive-striped flycatcher (*M. olivaceus*). Abbreviated candidate variables are sampling time of day, sampling date, molt status, hemoglobin concentration, relative body mass and sampling latency. Interspecific analyses employ body mass : tarsus ratio as the relative mass variable, whereas intraspecific analyses employ a scaled mass index. ^a^ ∆AIC_c_ = 0.060 (all other models listed in this table ranked first in AIC_c_ analyses)

**Fig. 2:**
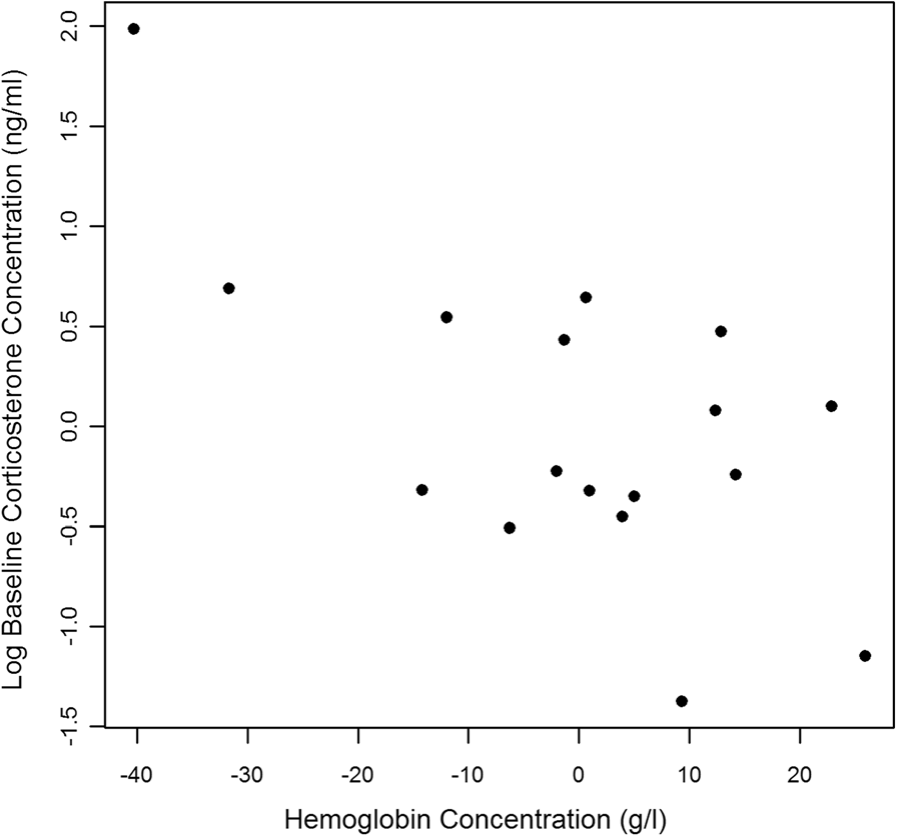
Partial regression plot for baseline corticosterone and hemoglobin concentration in the wedge-billed woodcreeper. Data are presented as unstandardized residuals after controlling for sampling time of day, which best explained baseline corticosterone alongside hemoglobin concentration in AIC_c_ model comparison (*r* ^2^ = 0.48, F_2,14_ = 6.389; see Table 1). Log denotes the natural logarithm (to base *e*)

Intraspecific model comparison identified hemoglobin concentration and sampling time of day as best predictors of baseline corticosterone in the wedge-billed woodcreeper (*r*^2^ = 0.48, F_2,14_ = 6.389), with higher baseline levels found in samples of lower hemoglobin content (Fig. 2) and in those collected earlier in the day. Substantial support was not found for any model of “increase in corticosterone” in this species. Only sampling date predicted “stress-induced corticosterone” (*r*^2^ = 0.27, F_1,15_ = 5.647), with individuals sampled later in the study period yielding lower levels 30 min post-capture.

No model for baseline corticosterone in the olive-striped flycatcher met the selection criteria. Analysis within this species identified scaled mass index as the best predictor of “increase in corticosterone” (*r*^2^ = 0.48, F_1,8_ = 7.440), with individuals indexed to higher relative mass yielding higher values for this corticosterone variable. This bivariate model of “increase in corticosterone” by scaled mass index remained significant for the “stress-induced corticosterone” variable (*r*^2^ = 0.47, F_1,8_ = 7.140).

Corticosterone values from the 41 species assessed in this study are summarized in suppl. Additional file 2: Table S2 with sample sizes and phylogeny. Capture times of day for all individuals are also reported in Additional file 3: Table S3.

## Discussion

### Diurnal variation in baseline corticosterone

Baseline corticosterone levels among 41 tropical passerine species sampled from free-living individuals generally replicated daily glucocorticoid rhythms detected in species-specific, within-individual repeated measures analyses of (primarily captive) temperate birds and mammals. As appears to be typical for mammals [4] and some non-passerines [6, 11, 56–58], diurnal baseline levels measured here were maximal at the onset of the active period and remained elevated hours thereafter. This does not oppose the dark-phase peak so far suggested for several temperate diurnal passerine species [12, 13, 16, 59], as birds were only assessed for corticosterone during daylight hours in this study. However, these results do not support the proposed rapid decline from a pre-active corticosterone peak to trough levels set early at the beginning of the light phase and maintained throughout the day [12–14].

Should avian corticosterone regulate caloric input, disposition and mobilization as do glucocorticoids in mammals [4], then our results’ asynchronous baseline nadir (relative to the passerine studies cited above) may indicate distinct patterns of diurnal energy intake and demand in tropical rainforest. This biome features high productivity, stable climatic regimes and complex trophic structure [60]. Given these features, birds may indeed follow different schedules of activity because starvation and predation risk are thought to modulate foraging patterns [61]. Under predictable conditions of low starvation (higher temperatures and reduced seasonality in the tropics) [62], foraging is predicted to become less concentrated to morning and shifted later into the day [63–65]. Such dispersed (less modal) foraging time is not predicted under high predation pressure [66, 67]. However, flock formation may substantially reduce predation risk (and perception) in tropical rainforest [68, 69]. The slowed transition to trough levels of baseline corticosterone observed in this study therefore fits multiple predictions of classical avian foraging theory. Relatively short day-length near the equator (at most ten foraging hours in the sub-canopy reduced further by rainfall and mid-day heat [70]) may also give rise to prolonged foraging [66] and baseline corticosterone elevation into late morning [62]. Photoperiod-dependent altitudinal variation in diurnal foraging cycles [71] and corticosterone release [8, 16] side with this notion. However, independent baseline release in several other cases [12, 13, 72] keep us from conclusions relating to photoperiod for this dataset.

In sum, our results adhere to a paradigm of time-dependent baseline corticosterone secretion that emphasizes feeding routine as a basis for diel patterns in the circulating levels of this glucocorticoid, given essential bidirectional interactions between adrenocortical output, activity and metabolism [4]. Variations do not appear to correspond to taxonomic differences *per se*, as others have proposed for passerines in reference to non-passerines [16] and mammals [8]. Rather, they may derive from distinctions in environmental conditions such as day-length, seasonality, productivity and predation risk.

### Additional correlates of baseline corticosterone

Apart from the negative correlation with sampling time of day, baseline corticosterone was also lower in molting birds of this study. This relationship persisted when analyses were conducted separately by sex and it conforms to the vast body of evidence for the down-regulation of baseline corticosterone during molt in passerines [24].

Baseline corticosterone also related to sampling date in male birds of this study. Higher levels measured at later sampling dates may follow the general progression of the breeding period into the post-hatching phase observed at our study site, during which provisioning of nestlings and fledglings likely predominates parental effort. Indeed, several recent studies present evidence for a positive effect of baseline corticosterone on foraging activity that promotes the supply of offspring with food to enhance reproductive success [73–75].

Hemoglobin concentration complemented sampling time of day as a negative correlate of baseline corticosterone in analysis within the wedge-billed woodcreeper. This negative relation reconciles poorly with other work examining links between hematocrit and corticosterone [76, 77]. However, elevated baseline corticosterone levels are often suggested to remedy poor body condition, in part by encouraging hyperphagia [78–80] when energetic status is compromised [81], such as when parents are feeding offspring. As a principal determinant of aerobic capacity, hemoglobin concentration robustly indicates energetic state [82] and is reduced when nutritional burden suppresses the formation of red blood cells [83]. It follows that low hemoglobin concentrations found in individuals of this study may have elicited the restitutive function of elevated baseline corticosterone introduced above. This perspective accommodates our data on birds captured primarily during breeding (i.e. compromised energetic condition). It also complies with theory that baseline corticosterone mediates trade-offs between parental investment and self-maintenance by regulating foraging activity [84].

Lastly, baseline corticosterone levels increased with sampling latency in interspecific analyses of samples taken within 3 min of capture. Initiation of stress-related corticosterone release has been specified to 1.5–2 min post-capture in other passerines before [85, 86]. Nevertheless, most studies continue to designate baseline levels based on the assumption that 3 min represent stress-independent release [48].

### Correlates of corticosterone release in response to stress

Evidence from the wild of time-dependent adrenocortical response to stress in birds remains elusive, as neither “increase in corticosterone” nor “stress-induced corticosterone” exhibited any diurnal rhythm in this study. To the contrary, our results confirm evidence for the down-regulation of the avian stress response during molt [24]. The negative interspecific “stress-induced corticosterone” relationship to molt status scaled up in analysis within males and also extended to the “increase in corticosterone” variable in this sex. Per definition (see Methods), “increase in corticosterone” is inversely related to baseline corticosterone levels. As such, its sustained correlation to molt status in the face of a parallel molt relationship at baseline levels emphasizes the weight of the molt effect on corticosterone release in response to stress. These results advocate the hypothesis of corticosterone down-regulation selected to preclude proteolytic action at stress-related concentrations during feather formation [87]. Such down-regulation should be particularly critical for molting males, for which plumage quality can come to dictate mating success [88].

“Increase in corticosterone” and “stress-induced corticosterone” also related positively to sampling date in our interspecific analyses of males, and a trend for the former was also present in females. As suggested for baseline corticosterone, these elevations may parallel the observed transition from incubation to post-hatching reproductive stages. Brooding effort in neo-tropical passerines often decreases with increased post-hatching provisioning (e.g. [89]) when offspring become more independent in thermoregulation but remain dependent for food acquisition [90] such that parents may afford a strong stress response to enhance short-term survival probability during increased foraging effort. Interestingly, sampling date correlated negatively with “stress-induced corticosterone” in the wedge-billed woodcreeper, a cavity-nesting bird [91]. Given lower levels of nest predation [92] and microclimate variation [93], cavity-nesters may shift high levels of nest attentiveness into later stages of the reproductive cycle [94] with associated alterations in the magnitude of their stress response.

Lastly, greater corticosterone release in response to stress was found in olive-striped flycatcher individuals with higher scaled mass indices. These indices indicate size of fat and protein reserves [52]. Their positive relation to “stress-induced corticosterone” (as well as the reduction of interspecific stress-related levels during molt, a process of high energetic cost [95]) therefore adds to accumulating evidence that an elevated energetic state is accompanied by greater stress-related corticosterone secretion [96–98]. This evidence sides with the hypothesis that the advantages of a strong adrenocortical response are most affordable when surplus fat and muscle substrates can be resigned to gluconeogenesis with little compromise to other vital physiological processes [99, 100].

## Conclusions

Our results indicate diurnal variation in baseline corticosterone release in free-living tropical forest birds. This rhythmicity appears robust enough to surface in analyses from observations pooled across a most diverse array of passerine species. It does not point to elementary departures from other taxa in temporal patterns and regulation of passerine corticosterone release. The observed baseline relationships presumably issue from bi-directional effects of corticosterone secretion and metabolic state as in other animals. Moreover, life-history state may set bounds on this condition-dependent corticosterone release at both baseline and stress-related levels. Modest alterations in corticosterone reaction to environmental and intrinsic signals are nevertheless likely to eventuate from ecological disparities among biomes of different latitudes. This study suggests certain responses to time of day and other variables to be tuned to life in high-productivity, relatively aseasonal tropical rainforest.

In practical terms, the results presented here offer several lines of orientation for improved methods in tropical avian field endocrinology. These include bearings for appropriate sampling schedule at multiple time scales (relating to season, time of day and latency) and variable parameterization (e.g. caution with relative measures of corticosterone response) as well as a range of corticosterone concentrations to be anticipated in tropical rainforest passerines. We hope that this information accelerates progress in grasping corticosterone’s complex interactions with avian physiology and behavior in the tropics and beyond.

## Ethics approval and consent to participate

This research was authorized by Ministerio del Ambiente del Ecuador in accordance to national guidelines.

## Consent for publication

Not applicable.

## Additional files

Additional file 1: Table S1. Baseline corticosterone (y1), “increase in corticosterone” (y2) and “stress-induced corticosterone” (y3) models from AIC_c_ analyses. (PDF 344 kb) Additional file 2: Table S2. Species median values with sample sizes for baseline and “stress-induced corticosterone” concentrations. (PDF 286 kb) Additional file 3: Table S3. Time of capture for 95 individuals (ID) of 41 species analyzed for baseline and stress-related corticosterone concentrations. (PDF 211 kb)
