# Supplementary material for: Diurnal variation in corticosterone release among wild tropical forest birds
Source: Front Zool. 2016 May 4;13:19. doi: 10.1186/s12983-016-0151-3 (PMC4857432; doi:10.1186/s12983-016-0151-3)
Supplement: Additional file 1: Table S1. — Baseline corticosterone (y1), “increase in corticosterone” (y2) and “stress-induced corticosterone” (y3) models from AICc analyses. (PDF 344 kb) [file 12983_2016_151_MOESM1_ESM.pdf]

Table S1. Baseline corticosterone (y_1), “increase in corticosterone” (y_2) and “stress-induced corticosterone” (y_3) models from AIC_c analyses.

	Response Variable	Linear Formula (Intercept and Variable Coefficients)							r^2	dF	F-ratio	ΔAIC_c	ω_i
		Int.	Time	Date	Molt	Hemo.	R. Mass	Lat.					
Total	**y_1**	**+1.871**	**-0.236**	**---**	**-0.325**	**---**	**---**	**+0.296**	**0.29**	**89**	**12.010**	**0.000**	**0.29**
	y_1	+1.873	-0.235	---	-0.332	+0.045	---	+0.304	0.29	88	9.063	1.816	0.12
	y_2	+4.004	---	+0.131	-0.310	---	---	---	0.07	89	3.568	0.000	0.12
	y_2	+4.004	---	+0.130	-0.313	---	+0.071	---	0.09	88	2.761	1.058	0.07
	y_2	+4.002	---	+0.122	-0.319	---	---	-0.070	0.08	88	2.694	1.251	0.06
	y_2	+3.985	---	---	-0.263	---	---	---	0.04	90	3.385	1.513	0.05
	**y_3**	**+4.130**	**---**	**---**	**-0.310**	**---**	**---**	**n/a**	**0.06**	**93**	**5.526**	**0.000**	**0.19**
	y_3	+4.139	---	+0.072	-0.337	---	---	n/a	0.07	92	3.394	0.901	0.12
	y_3	+4.131	---	---	-0.313	---	+0.058	n/a	0.06	92	3.186	1.303	0.10
	y_3	+4.141	-0.040	---	-0.343	---	---	n/a	0.06	92	2.908	1.841	0.08
Males	**y_1**	**+2.179**	**-0.312**	**+0.377**	**-0.714**	**---**	**---**	**+0.278**	**0.49**	**25**	**6.044**	**0.000**	**0.26**
	**y_2**	**+4.303**	**---**	**+0.376**	**-0.838**	**---**	**---**	**---**	**0.27**	**27**	**4.949**	**0.000**	**0.24**
	y_2	+4.303	---	+0.373	-0.858	---	---	-0.151	0.31	26	3.804	1.354	0.12
	**y_3**	**+4.396**	**---**	**+0.378**	**-0.757**	**---**	**---**	**n/a**	**0.26**	**28**	**4.971**	**0.000**	**0.36**
Females	y_1	+1.828	-0.293	---	-0.600	---	---	+0.227	0.36	25	4.630	0.000	0.09
	**y_1**	**+1.838**	**-0.356**	**---**	**-0.661**	**---**	**---**	**---**	**0.29**	**26**	**5.234**	**0.060**	**0.09**
	y_1	+1.870	-0.292	---	-0.695	+0.214	---	---	0.42	24	4.344	0.229	0.08
	y_1	+1.881	-0.354	---	-0.756	+0.214	---	---	0.35	25	4.484	0.327	0.08
	y_1	+1.799	-0.304	---	-0.560	---	+0.203	+0.282	0.40	24	4.032	1.117	0.05
	y_1	+1.866	---	---	-0.489	---	---	+0.302	0.25	26	4.311	1.566	0.04
	y_1	+1.908	---	---	-0.585	+0.216	---	+0.302	0.31	25	3.796	1.928	0.03
	y_2	+3.731	---	+0.225	---	---	---	---	0.14	26	4.058	0.000	0.19
	y_2	+3.841	---	+0.262	-0.282	---	---	---	0.18	25	2.672	1.375	0.09
	y_2	+3.725	---	+0.224	---	---	+0.111	---	0.16	25	2.338	2.000	0.07
	y_3	+4.022	---	---	-0.395	---	---	n/a	0.10	28	2.977	0.000	0.20
	y_3	+4.041	---	+0.120	-0.472	---	---	n/a	0.13	27	2.096	1.379	0.10

Table S1 (Continued).

	Response Variable	Linear Formula (Intercept and Variable Coefficients)							r^2	dF	F-ratio	ΔAIC_c	ω_i
		Int.	Time	Date	Molt	Hemo.	R. Mass	Lat.					
G. spirurus	**y_1**	**+1.952**	**-0.667**	**---**	**---**	**-0.713**	**---**	**---**	**0.47**	**14**	**6.389**	**0.000**	**0.36**
	y_2	+3.841	---	---	-0.814	---	-0.307	---	0.35	13	3.522	0.000	0.13
	y_2	+3.739	---	---	-0.595	---	---	---	0.18	14	3.141	0.052	0.12
	y_2	+3.402	+0.281	---	---	---	---	---	0.14	14	2.208	0.947	0.08
	y_2	+3.392	---	-0.302	---	---	---	---	0.13	14	2.127	1.027	0.08
	**y_3**	**+3.525**	**---**	**-0.439**	**---**	**---**	**---**	**n/a**	**0.27**	**15**	**5.647**	**0.000**	**0.21**
	y_3	+3.524	---	-0.462	---	---	-0.205	n/a	0.34	14	3.674	1.746	0.09
	y_3	+3.749	---	-0.366	-0.412	---	---	n/a	0.34	14	3.640	1.801	0.09
M. olivaceus	y_1	+1.999	-0.321	---	---	---	---	---	0.26	8	2.741	0.000	0.22
	y_1	+1.857	---	---	---	---	---	+0.362	0.22	8	2.201	0.516	0.17
	y_1	+1.780	---	---	+0.603	---	---	---	0.15	8	1.371	1.364	0.11
	y_1	+1.765	-0.470	---	---	+0.447	---	---	0.53	7	3.882	1.483	0.11
	**y_2**	**+4.510**	**---**	**---**	**---**	**---**	**+0.366**	**---**	**0.48**	**8**	**7.440**	**0.000**	**0.51**
	**y_3**	**+4.601**	**---**	**---**	**---**	**---**	**+0.346**	**n/a**	**0.47**	**8**	**7.140**	**0.000**	**0.54**

Models are listed with formula, coefficient of determination (r^2), degrees of freedom (dF), F-ratio, ΔAIC_c and Akaike weight (ω_i). Selected models (indicated in bold) are within 2 AIC_c of top-ranked models. The credible intervals of their predictor coefficients do not overlap zero. Subordinate models meet only the former of these 2 selection criteria. Corticosterone response variables (ng/ml) are log-transformed to base *e*. Models are given for total and sex-partitioned interspecific corticosterone variation as well as for intraspecific analyses of the wedge-billed woodcreeper (*G. spirurus*) and olive-striped flycatcher (*M. olivaceus*). Abbreviated candidate variables are sampling time of day, sampling date, molt status, hemoglobin concentration, relative body mass and sampling latency. Interspecific analyses employ body mass : tarsus ratio as the relative mass variable, whereas intraspecific analyses employ a scaled mass index.
