# Supplementary material for: Diurnal variation in corticosterone release among wild tropical forest birds
Source: Front Zool. 2016 May 4;13:19. doi: 10.1186/s12983-016-0151-3 (PMC4857432; doi:10.1186/s12983-016-0151-3)
Supplement: Additional file 2: Table S2. — Species median values with sample sizes for baseline and “stress-induced corticosterone” concentrations. (PDF 286 kb) [file 12983_2016_151_MOESM2_ESM.pdf]

Table S2. Species median values with sample sizes for baseline and "stress-induced corticosterone" concentrations.

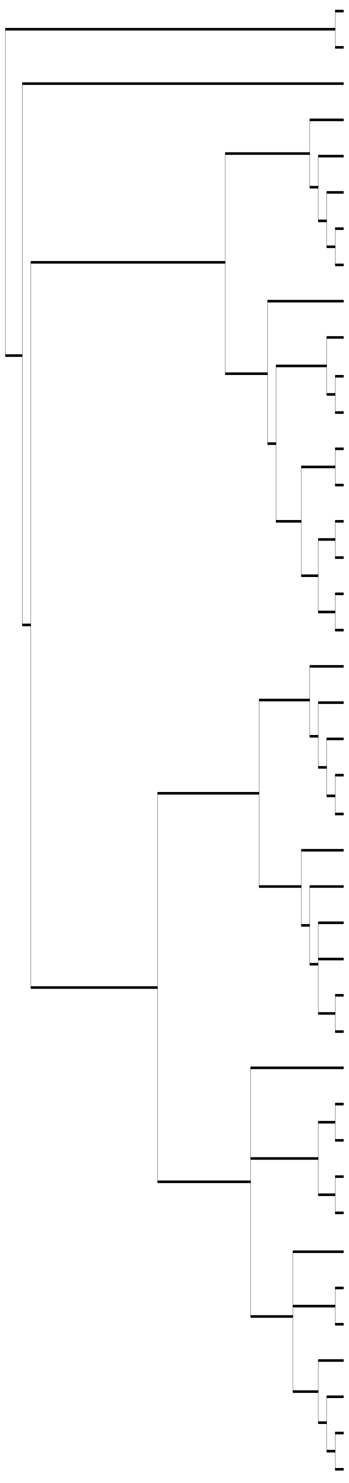

Species	Median Corticosterone Concentration (ng/ml)		n
	Baseline	Stress-induced	
Capito squamatus	10.756	99.764	1
Galbula ruficauda	6.841	54.248	1
Pyrrhura melanura	2.770	32.144	1
Myadestes ralloides	4.208	55.552	1
Microbates cinereiventris	2.861	42.858	4
Microcerculus marginatus	2.605	37.458	1
Henicorhina leucosticta	14.776	66.031	1
Henicorhina leucophrys	6.386	46.636	3
Euphonia xanthogaster	4.184	43.267	6
Chlorothraupis stolzmanni	3.026	47.537	1
Tachyphonus delatrii	11.039	19.120	1
Oryzoborus funereus	7.792	44.490	1
Arremon aurantiirostris	5.723	79.685	2
Arremon brunneinucha	3.014	52.349	1
Basileuterus chrysogaster	9.923	63.403	2
Myioborus miniatus	15.033	63.458	1
Basileuterus tristriatus	4.811	53.955	1
Phaeothlypis fulvicauda	5.229	49.835	1
Myrmotherula schisticolor	1.484	58.780	2
Gymnopithys leucaspis	9.351	96.503	2
Myrmeciza immaculata	2.766	37.319	2
Myrmeciza nigricauda	5.075	107.348	1
Myrmeciza exsul	23.996	108.341	1
Sclerurus mexicanus	2.681	13.494	1
Premnoplex brunnescens	5.526	92.870	2
Glyphorynchus spirurus	4.695	34.776	17
Dendrocincla fuliginosa	5.381	104.342	2
Dendrocolaptes sanctithomae	7.552	69.457	1
Xiphorhynchus erythropygius	2.132	114.750	1
Snowornis cryptolophus	5.799	112.036	1
Machaeropterus deliciosus	5.076	82.620	4
Manacus manacus	13.831	78.789	1
Xenopipo holochlora	6.082	77.007	1
Masius chrysopterus	11.254	64.235	2
Schiffornis turdina	4.551	108.496	1
Myiobius sulphureipygius	5.039	51.005	3
Myiobius villosus	2.027	53.742	3
Rhynchocyclus pacificus	1.630	48.053	1
Pseudotriccus pelzelni	10.499	77.839	1
Leptopogon superciliaris	10.854	69.574	5
Mionectes olivaceus	6.176	98.332	10

Corticosterone levels were measured from a standard 120 min capture and handling protocol. Phylogenetic relationships depicted in the consensus tree at left were established from recent avian molecular phylogenies [55].
