# Supplementary material for: Diurnal variation in corticosterone release among wild tropical forest birds
Source: Front Zool. 2016 May 4;13:19. doi: 10.1186/s12983-016-0151-3 (PMC4857432; doi:10.1186/s12983-016-0151-3)
Supplement: Additional file 3: Table S3. — Time of capture for 95 individuals (ID) of 41 species analyzed for baseline and stress-related corticosterone concentrations. (PDF 211 kb) [file 12983_2016_151_MOESM3_ESM.pdf]

Table S3. Time of capture for 95 individuals (ID) of 41 species analyzed for baseline and stress-related corticosterone concentrations.

ID	Species	Time of Capture
A86	*Myrmotherula schisticolor*	6:09
A100	*Basileuterus chrysogaster*	6:15
A96	*Euphonia xanthogaster*	7:01
B54	*Glyphorynchus spirurus*	7:15
A19	*Masius chrysopterus*	7:21
A101	*Gymnopithys leucaspis*	7:24
B26	*Xenopipo holochlora*	7:53
A92	*Microbates cinereiventris*	7:53
B60	*Gymnopithys leucaspis*	7:57
B77	*Rhynchocyclus pacificus*	7:58
B6	*Oryzoborus funereus*	8:05
A94	*Microbates cinereiventris*	8:06
A97	*Euphonia xanthogaster*	8:07
B4	*Mionectes olivaceus*	8:08
A83	*Leptopogon superciliaris*	8:08
B51	*Myrmeciza exsul*	8:10
B17	*Arremon aurantiirostris*	8:23
B37	*Myrmeciza immaculata*	8:29
B64	*Euphonia xanthogaster*	8:33
B71	*Glyphorynchus spirurus*	8:34
B42	*Euphonia xanthogaster*	8:39
B41	*Euphonia xanthogaster*	8:39
B72	*Glyphorynchus spirurus*	8:42
A36	*Glyphorynchus spirurus*	8:47
A50	*Glyphorynchus spirurus*	8:49
A84	*Henicorhina leucophrys*	8:52
A102	*Basileuterus chrysogaster*	8:55
A63	*Machaeropterus deliciosus*	8:58
A56	*Glyphorynchus spirurus*	9:02
B18	*Manacus manacus*	9:19
B33	*Henicorhina leucosticta*	9:21
B78	*Mionectes olivaceus*	9:22
A87	*Dendrocincla fuliginosa*	9:23
A28	*Glyphorynchus spirurus*	9:26
B67	*Capito squamatus*	9:33
B12	*Myiobius sulphureipygius*	9:35
B28	*Mionectes olivaceus*	9:38
B55	*Dendrocincla fuliginosa*	9:49
A3	*Basileuterus tristriatus*	9:50

Table S3 (Continued).

ID	Species	Time of Capture
A93	*Henicorhina leucophrys*	10:00
A88	*Machaeropterus deliciosus*	10:03
A61	*Myrmeciza nigricauda*	10:07
B34	*Mionectes olivaceus*	10:13
B10	*Tachyphonus delatrii*	10:13
B23	*Microbates cinereiventris*	10:34
B7	*Mionectes olivaceus*	10:45
B68	*Galbula ruficauda*	10:56
A6	*Pseudotriccus pelzelni*	11:05
A49	*Glyphorynchus spirurus*	11:11
A51	*Masius chrysopterus*	11:14
A17	*Chlorothraupis stolzmanni*	11:15
A41	*Myrmotherula schisticolor*	11:16
A65	*Myiobius villosus*	11:19
B43	*Pyrrhura melanura*	11:30
A80	*Myadestes ralloides*	11:35
A59	*Premnoplex brunnescens*	11:39
A64	*Henicorhina leucophrys*	11:43
A81	*Glyphorynchus spirurus*	11:50
A82	*Glyphorynchus spirurus*	11:56
B38	*Mionectes olivaceus*	11:58
A15	*Sclerurus mexicanus*	12:11
A104	*Leptopogon superciliaris*	12:11
A105	*Leptopogon superciliaris*	12:11
B48	*Glyphorynchus spirurus*	12:27
B39	*Myrmeciza immaculata*	12:32
A42	*Premnoplex brunnescens*	12:34
B27	*Myiobius sulphureipygius*	12:35
B76	*Microbates cinereiventris*	12:35
A53	*Myiobius villosus*	12:42
A25	*Glyphorynchus spirurus*	12:43
B74	*Mionectes olivaceus*	12:43
B61	*Schiffornis turdina*	12:50
B66	*Leptopogon superciliaris*	13:08
A69	*Glyphorynchus spirurus*	13:33
B29	*Euphonia xanthogaster*	13:35
B52	*Glyphorynchus spirurus*	13:44
A38	*Machaeropterus deliciosus*	13:58
B16	*Glyphorynchus spirurus*	14:14
A30	*Microcerculus marginatus*	14:21

Table S3 (Continued).

ID	Species	Time of Capture
B53	*Myiobius sulphureipygius*	14:40
B13	*Glyphorynchus spirurus*	14:43
B5	*Leptopogon superciliaris*	14:49
B45	*Machaeropterus deliciosus*	14:51
B30	*Xiphorhynchus erythropygius*	14:55
B35	*Mionectes olivaceus*	15:00
A98	*Myiobius villosus*	15:09
B31	*Dendrocolaptes sanctithomae*	15:19
B36	*Mionectes olivaceus*	15:25
B40	*Glyphorynchus spirurus*	15:44
A99	*Myioborus miniatus*	16:01
A47	*Arremon brunneinucha*	16:02
A52	*Snowornis cryptolophus*	16:02
B50	*Phaeothlypis fulvicauda*	16:06
B19	*Arremon aurantiirostris*	16:12
B32	*Mionectes olivaceus*	16:23
